# Intracellular fluid accumulation underlies brain volume increases in early Alzheimer’s disease

**DOI:** 10.1093/braincomms/fcag075

**Published:** 2026-03-10

**Authors:** Michalis Kassinopoulos, Paula Montesinos, Carles Falcon, Jordi Huguet, Carolina Minguillon, Karine Fauria, Gwendlyn Kollmorgen, Clara Quijano-Rubio, José Luis Molinuevo, Oriol Grau-Rivera, Henrik Zetterberg, Kaj Blennow, Marc Suárez-Calvet, Javier Sanchez-Gonzalez, Juan Domingo Gispert, ALFA study, Müge Akinci, Federica Anastasi, Annabella Beteta, Raffaele Cacciaglia, Lidia Canals, Alba Cañas, Carme Deulofeu, Maria Emilio, Irene Cumplido-Mayoral, Marta del Campo, Carme Deulofeu, Ruth Dominguez, Maria Emilio, Sherezade Fuentes, Marina García, Laura Hernández, Gema Huesa, Laura Iglesias, Esther Jiménez, David López-Martos, Paula Marne, Tania Menchón, Paula Ortiz-Romero, Eleni Palpatzis, Wiesje Pelkmans, Albina Polo, Sandra Pradas, Mahnaz Shekari, Lluís Solsona, Anna Soteras, Núria Tort-Colet, Marc Vilanova, Natalia Vilor Tejedor

**Affiliations:** Barcelonaβeta Brain Research Center (BBRC), Pasqual Maragall Foundation, Barcelona 08005, Spain; Philips Healthcare Iberia, Madrid 28050, Spain; Barcelonaβeta Brain Research Center (BBRC), Pasqual Maragall Foundation, Barcelona 08005, Spain; Centro de Investigación Biomédica en Red de Bioingeniería, Biomateriales y Nanomedicina (CIBER-BBN), Instituto de Salud Carlos III, Madrid 28029, Spain; Hospital del Mar Research Institute, Barcelona 08003, Spain; Barcelonaβeta Brain Research Center (BBRC), Pasqual Maragall Foundation, Barcelona 08005, Spain; Barcelonaβeta Brain Research Center (BBRC), Pasqual Maragall Foundation, Barcelona 08005, Spain; Hospital del Mar Research Institute, Barcelona 08003, Spain; Barcelonaβeta Brain Research Center (BBRC), Pasqual Maragall Foundation, Barcelona 08005, Spain; Hospital del Mar Research Institute, Barcelona 08003, Spain; Centro de Investigación Biomédica en Red de Fragilidad y Envejecimiento Saludable (CIBERFES), Instituto de Salud Carlos III, Madrid 28029, Spain; Roche Diagnostics GmbH, Penzberg 82377, Germany; Roche Diagnostics International Ltd, Rotkreuz 6343, Switzerland; Barcelonaβeta Brain Research Center (BBRC), Pasqual Maragall Foundation, Barcelona 08005, Spain; Barcelonaβeta Brain Research Center (BBRC), Pasqual Maragall Foundation, Barcelona 08005, Spain; Hospital del Mar Research Institute, Barcelona 08003, Spain; Centro de Investigación Biomédica en Red de Fragilidad y Envejecimiento Saludable (CIBERFES), Instituto de Salud Carlos III, Madrid 28029, Spain; Servei de Neurologia, Hospital del Mar, Barcelona 08003, Spain; Clinical Neurochemistry Laboratory, Sahlgrenska University Hospital, Mölndal 431 80, Sweden; Department of Psychiatry and Neurochemistry, Institute of Neuroscience and Physiology, The Sahlgrenska Academy at University of Gothenburg, Mölndal 431 80, Sweden; UK Dementia Research Institute at University College London (UCL), London WC1N 3BG, UK; Department of Neurodegenerative Disease, UCL Institute of Neurology, London WC1M 3BG, UK; Hong Kong Center for Neurodegenerative Diseases, Hong Kong, China; Wisconsin Alzheimer’s Disease Research Center, University of Wisconsin School of Medicine and Public Health, University of Wisconsin-Madison, Madison 53705, WI, USA; Clinical Neurochemistry Laboratory, Sahlgrenska University Hospital, Mölndal 431 80, Sweden; Department of Psychiatry and Neurochemistry, Institute of Neuroscience and Physiology, The Sahlgrenska Academy at University of Gothenburg, Mölndal 431 80, Sweden; Paris Brain Institute, ICM, Pitié-Salpêtrière Hospital, Sorbonne University, Paris 75013, France; Neurodegenerative Disorder Research Center, Division of Life Sciences and Medicine, and Department of Neurology, Institute on Aging and Brain Disorders, University of Science and Technology of China and First Affiliated Hospital of USTC, Hefei 230001, P.R. China; Barcelonaβeta Brain Research Center (BBRC), Pasqual Maragall Foundation, Barcelona 08005, Spain; Hospital del Mar Research Institute, Barcelona 08003, Spain; Centro de Investigación Biomédica en Red de Fragilidad y Envejecimiento Saludable (CIBERFES), Instituto de Salud Carlos III, Madrid 28029, Spain; Servei de Neurologia, Hospital del Mar, Barcelona 08003, Spain; Philips Healthcare Iberia, Madrid 28050, Spain; Barcelonaβeta Brain Research Center (BBRC), Pasqual Maragall Foundation, Barcelona 08005, Spain; Centro de Investigación Biomédica en Red de Bioingeniería, Biomateriales y Nanomedicina (CIBER-BBN), Instituto de Salud Carlos III, Madrid 28029, Spain; Hospital del Mar Research Institute, Barcelona 08003, Spain

**Keywords:** neuroinflammation, brain volume, early AD, multi-shell DWI, amyloid PET

## Abstract

In the preclinical stages of Alzheimer’s disease, increased brain volume has been associated with amyloid-beta pathology, particularly in regions that undergo volume reductions as the disease progresses. Glial reactivity and water diffusion alterations have been linked to such macroscopic volumetric changes. Brain volume reductions have also been reported following amyloid-beta removal with anti-amyloid therapies with beneficial clinical effects, but it remains unclear whether these changes result from resolving amyloid-triggered neuroinflammation or neurodegeneration. Intravoxel incoherent motion modelling based on multi-shell diffusion-weighted imaging may provide a better understanding of the processes underlying these paradoxical changes. This study used intravoxel incoherent motion diffusion MRI to examine how alterations in cerebral water pools contribute to increased brain volume linked to amyloid-beta deposition and neuroinflammation in cognitively unimpaired individuals. We developed a three-compartment diffusion MRI model with four parameters of cerebral water diffusion: slow diffusion coefficient, fast diffusion coefficient, slow signal portion, and perfusion fraction. We computed these diffusion parameters in 297 cognitively unimpaired late middle-aged adults, 35% of whom showed evidence of amyloid deposition. We examined their correlation with demographic factors (age, sex, apolipoprotein E status), markers of Alzheimer’s disease pathology, neurodegeneration, neuroinflammation, and mean diffusivity. Then, we identified regions showing grey matter volume increases related to amyloid burden and examined the association between grey matter volume and diffusion parameters within these regions. We did not find evidence of associations between diffusion parameters and amyloid-related biomarkers, whether assessed by PET or cerebrospinal fluid measures. In contrast, the four diffusion parameters showed strong and widespread associations with biomarkers of neuroinflammation and neurodegeneration, particularly in frontoparietal and cingulate regions. Additionally, in grey matter regions where volume increases were related to amyloid levels, volumes were negatively correlated with the slow diffusion coefficient (*P* = 0.001), perfusion fraction (*P* = 0.036) and mean diffusivity (*P* = 0.047). These findings indicate that diffusion-derived measures are more sensitive to neuroinflammatory and neurodegenerative processes than to amyloid pathology in cognitively unimpaired individuals. Furthermore, the observed negative association between grey matter volume and slow diffusion coefficient in amyloid-related regions may reflect increased cellular complexity rather than intracellular water accumulation. This interpretation suggests that glial remodelling or microstructural changes could underlie brain volume increases in amyloid-positive individuals without overt neurodegeneration. These results underscore the value of intravoxel incoherent motion-derived metrics for gaining deeper insights into the pathophysiological mechanisms of Alzheimer’s disease, influencing brain volume changes as well as those resulting from the response to anti-amyloid therapies.

## Teaser

Kassinopoulos *et al*. examine how amyloid-β pathology in cognitively unimpaired individuals is linked to regional grey matter volume increases. Using diffusion MRI, they show that reduced intracellular water diffusion, likely reflecting neuroinflammation-related increased cellular complexity or glial remodelling, may underlie these volumetric changes.

## Graphical Abstract

**Graphical Abstract:**
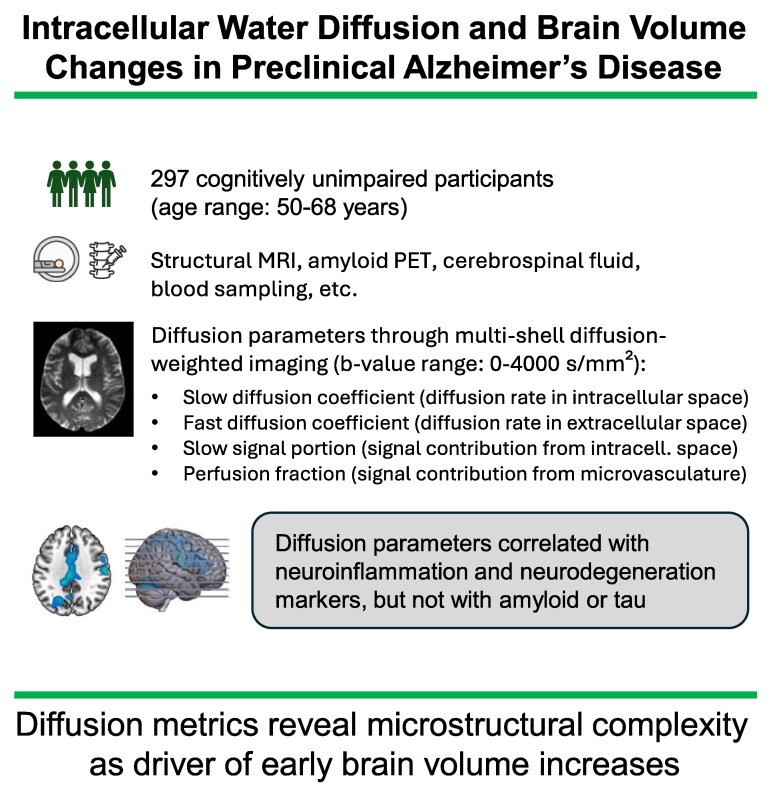

## Introduction

Alzheimer’s disease (AD) is a neurodegenerative disease characterized by the accumulation of amyloid beta (Aβ) plaques and tau neurofibrillary tangles.^1^ Typically, Aβ accumulates in the brain in late-middle-aged individuals who can remain cognitively unimpaired for decades. At these early preclinical stages, increases in brain volume have been linked with a neuroinflammatory response to Aβ accumulation and changes in water diffusion.^2,3^ Neuroinflammation is a complex response to cerebral tissue injury involving the activation of immunocompetent cells -microglia, astrocytes- and can lead to brain swelling due to the accumulation of fluid in the tissue. In particular, increases in brain volume have been linked to an increase in CSF neuroinflammation biomarkers: soluble triggering receptor expressed on myeloid cells 2 (sTREM2), S100 calcium-binding protein (S100B), chitinase-3-like protein 1 (YKL-40), interleukin 6 (IL-6), and glial fibrillary acidic protein (GFAP)^4-7^ Moreover, an increase in CSF neuroinflammation biomarkers has been associated with reductions in cerebral water diffusion in preclinical and symptomatic AD stages.^8^

Recently, Aβ removal through the use of monoclonal antibodies has been shown to be clinically beneficial in patients with mild cognitive decline and mild dementia due to AD.^9,10^ However, paradoxically, such Aβ removal has also been associated with a reduction in brain volume, which is typically interpreted as a sign of accelerated neurodegeneration.^11^ Yet, previous findings suggest that the reduction in brain volume with amyloid removal could be due to amyloid plaque clearance and associated cerebral fluid shifts,^12^ as well as due to the resolution of the neuroinflammatory response to Aβ.^13^ Given that neuroinflammation and cerebral water accumulation are closely tied to AD pathophysiology, exploring the relationships between distinct cerebral water pools, neuroinflammatory activity, and brain volume may offer insights into the mechanisms underlying the paradoxical brain volume reductions observed following anti-amyloid monoclonal antibody treatment in early symptomatic AD.

In this study, we aimed to gain a better understanding of the relationship between cerebral water diffusion, neuroinflammation, and brain volume in a late middle-aged cohort of cognitively unimpaired individuals, many with positive Aβ biomarkers. Using multi-shell Diffusion-Weighted Imaging (DWI) combined with a three-compartment intravoxel incoherent motion (IVIM) model,^14^ we derived four diffusion-related parameters that distinguish contributions from the intracellular and extracellular space. Two of these parameters reflect the rate of diffusion in each space, while the other two reflect the relative signal contributions from the intracellular water pool and the microvasculature. We first analysed the possible association of these parameters with (a) participant characteristics such as age, sex, and apolipoprotein E (*APOE*) genotype; (b) biomarkers of AD (amyloid-PET positivity, and Aβ and phosphorylated tau proteins from the CSF) and neurodegeneration (neurofilament light [NfL] from the CSF, hippocampal volume, and AD signature [average cortical thickness in a set of brain regions known to undergo atrophy in AD])^15,16^; (c) biomarkers of neuroinflammation (sTREM2, S100B, YKL-40, IL6, and GFAP from the CSF, and plasma GFAP); and (d) mean diffusivity (MD). Then, we examined regions with GM volume increases related to amyloid load and assessed the associations between IVIM parameters, MD, and GM volume in these regions. These analyses aimed to explore the potential contribution of cerebral water diffusion to the increase in GM volume, with a particular focus on neuroinflammatory processes.

## Materials and methods

### Study sample

This was an observational, cross-sectional, case-control study that included 297 late middle-aged adults from the ALFA + cohort.^17^ All ALFA + participants were cognitively unimpaired with a Mini-Mental State Examination above 26 and a Clinical Dementia Rating of 0. They were tested for the *APOE-ε4* allele, and most had a family history of AD.^18^ All subjects gave written informed consent according to the Declaration of Helsinki, and the study was approved by the independent Ethics Committee ‘Parc de Salut Mar’ Barcelona (Spain).

**Figure 1:**
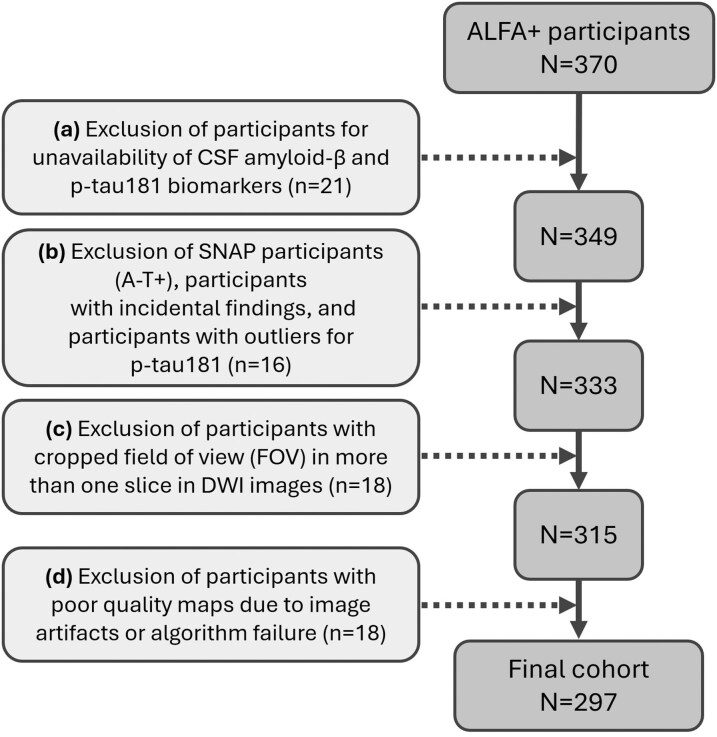
**Participant characteristics flow chart.** Flow chart of the participant selection. A total of 370 ALFA + participants underwent multi-shell DWI. Of these, 73 were excluded for the following reasons: (**A**) unavailability of CSF amyloid-β and p-tau181 biomarkers (*n* = 21); (**B**) evidence of Suspected Non-Alzheimer’s disease Pathophysiology (SNAP, i.e. A − T+), incidental findings, or outliers for p-tau181 values exceeding five standard deviations from mean (*n* = 16); (**C**) cropped field of view (FOV) in acquired DWI images for more than one slice (*n* = 18); and (**D**) poor quality maps related to image artifacts or algorithm failure (*n* = 18). Based on this selection, 297 participants were considered in the present study.

Figure 1 summarizes the participant selection process and exclusion criteria of the 370 participants who underwent MRI acquisition for the ALFA + study. Out of these, 73 participants were excluded due to reasons mentioned in Fig. 1, leaving 297 participants in the present study. Aβ PET, *APOE* genotyping, and fluid sampling and biomarker analyses are described in the Supplementary Materials.

### Structural MRI acquisition

Data were acquired using a 3T Philips Ingenia CX scanner at the neuroimaging unit at the Barcelonaβeta Brain Research Center using a 32-channel head coil. High-resolution structural images were acquired using a 3D T1-weighted (T1w) Turbo Field Echo pulse sequence (TR/TE/TI = 9.9/4.6/900 ms, flip angle = 8°, FOV = 240 × 240 × 180 mm^3^, sagittal orientation, voxel size = 0.75 × 0.75 × 0.75 mm^3^).

### DWI acquisition

Multi-shell DWI images were acquired using a multi-slice single-shot Spin Echo-Planar Imaging (EPI) sequence (TR = 5000 ms, FOV = 240 × 240 × 128 mm^3^, acquired voxel size = 2.0 × 2.0 × 4.0 mm^3^, reconstructed voxel size = 0.94 × 0.94 × 4.0 mm^3^). The acquisition was split into three sequences with different *b*-values to evaluate water diffusion within different compartments of the brain. First sequence: TE = 42 ms, *b* = 0, 150 s/mm^2^; second sequence: TE = 60 ms, *b* = 0, 300, 500, 1000 s/mm^2^; and third sequence: TE = 84 ms, *b* = 0, 2000, 4000 s/mm^2^. The total acquisition time was around four minutes and 15 s. Additionally, single-shell DWI images were acquired using a pulse sequence optimized for diffusion tensor imaging (DTI) with the following parameters: 64 unique diffusion-encoding directions (b = 1300 s/mm^2^), TR = 9000 ms, TE = 90 ms, FOV = 230 × 230 × 145 mm^3^, and voxel size = 2.1 × 2.1 × 2.2 mm. These single-shell images were used to compute MD maps, which are commonly employed in diffusion MRI studies.

### Structural MRI quantifications

Hippocampal volume was measured as a marker of neurodegeneration. We segmented the hippocampus on T1w MRI images using FreeSurfer version 7.1.1. We then constructed a bilateral hippocampal volume by summing up the volumes in the left and right hemispheres. Finally, we employed linear regression analysis to remove the effect of total intracranial volume (TIV), derived from FreeSurfer version 7.1.1, and obtain the TIV-adjusted hippocampal volume.^19^

AD signature was also measured as a marker of neurodegeneration. We computed the Jack *et al*. AD-signature composite^15^ from T1w images using FreeSurfer version 7.1.1. This measure is based on the surface-area weighted average of the mean cortical thickness in entorhinal, inferior temporal and middle temporal cortices, and the fusiform gyri.^16^

Maps of modulated grey matter (GM) volumes were obtained using the DARTEL implementation for voxel-based morphometry in SPM12. These maps were then smoothed with a 12 mm full-width at half maximum (FWHM) Gaussian kernel.

### DWI quantifications

We derived four water diffusion parameters from a three-compartment IVIM DWI model, referred to later as IVIM parameters. This model assumes three intravoxel compartments, as described in Equation (1):


(1) $$S=S_{0}\left[\;fe^{-TE/T2_{v}}e^{D^{*}b}+(1-f)e^{-TE/T2_{t}}\;F_{t}(D_{f},\;D_{s})\right]$$

The first compartment represents the fast motion of the water pool inside blood vessels and is determined by the information provided by diffusion signal decay at small *b*-values. The proportion of signal drop due to water movement inside the vessels is represented by the parameter *f* (perfusion fraction) and the diffusion coefficient for this compartment is represented by D*.

The second and third compartments correspond to the extra- and intracellular spaces, respectively. The intracellular water diffusivity is slower than the extracellular one due to cellular organelles. These two compartments are represented in Equation (1) by $F_{t}(D_{f},\;D_{s})$ that is modelled using Equation (2):


(2) $$F_{t}(D_{f},\;D_{s})=\;[(1-SSP)e^{-D_{f}\;b}+SSP\;e^{-D_{s}b}]$$

The parameter $D_{f}$ represents the diffusion coefficient for the signal decay due to the diffusion of extracellular water (fast DC), and $D_{s}$ represents the diffusion coefficient for the signal decay due to the diffusion of intracellular water (slow DC). The *SSP* parameter represents the slow signal portion inside the voxel. Finally, in Equation (1), $T2_{v}\;$ and $T2_{t}$ represent the T2 relaxation parameters in the vascular and tissue compartments.

### Diffusion signal analysis

Before modelling, the images from the different acquisitions were registered using affine registration to correct signal distortions and/or patient movement during the acquisitions. A first set of initial values for the model was obtained from the different images acquired at *b* = 0 s/mm^2^, which were used to estimate T2 values. Later, the different diffusion coefficients were estimated in reverse order, from high *b-*values to estimate $D_{s}$, to intermediate *b*-values to estimate $D_{f}$. For the perfusion component, only *f* was estimated using a simplified IVIM model.^20^

Once the initial values were obtained, a final non-linear fitting was performed with the whole model to ensure better accuracy and signal coherence between different compartments. The whole pipeline was implemented using Interactive Data Language (IDL), producing the four IVIM parameters ($D_{s},\;D_{f},\;SSP,$ and f) and the corresponding maps. These maps were normalized to the Montreal Neurological Institute (MNI) space through non-linear registration using the SPM12 software.

### Correlation between mean diffusivity (MD) and the three-compartment IVIM parameters

To better understand what the four parameters of our three-compartment IVIM model reflect, we compared them with the parameter MD, derived from the single-shell DWI acquisition. The DWI images were corrected for magnetic field inhomogeneity distortions (TOPUP) and eddy current distortions using the FMRIB Software Library (FSL) software package,^21^ and MD maps were obtained with the FSL tool DTIFIT. The MD maps were then normalized to the Montreal Neurological Institute (MNI) space through non-linear registration using the SPM12 software. The correspondence between MD maps and maps of our three-compartment IVIM parameters was assessed within a mask consisting of GM and white matter (WM) voxels using Pearson correlation.

### Statistical analysis

We first sought the association of our four IVIM parameters across voxels in the whole brain with age, sex, and *APOE*-ε4 in individuals with normal core AD biomarkers (i.e. subjects without accumulation of Aβ and tau proteins, A − T − group). We then examined voxel-wise associations between GM volume maps, obtained using DARTEL, and each of the IVIM parameters across all individuals. All analyses were adjusted for age, sex, and *APOE*-ε4 status. As these analyses revealed strong associations, GM volume was regressed out from all IVIM parameter maps prior to conducting subsequent statistical analyses in the entire cohort, to account for its potential confounding influence.

Following this adjustment, we examined differences in our IVIM parameters between subjects either with an accumulation of Aβ and not tau proteins (A + T − group), or an accumulation of Aβ and tau proteins (A + T + group), and the A − T − control group. We also examined the relationship of the proposed IVIM parameters with binary amyloid-PET positivity (>12CL) and continuous measures of AD biomarkers (Aβ42/Aβ40 and p-tau181/Aβ40 from the CSF), neurodegeneration biomarkers (CSF NfL, AD signature, and hippocampal volume), and neuroinflammation biomarkers (sTREM2, S100B, YKL-40, IL6, and GFAP from the CSF, and plasma GFAP), in all individuals. We used the p-tau181 values for assessing tau positivity based on the cut-offs proposed by Milà-Alomà *et al.*.^22^ We normalized all CSF biomarkers to Aβ40 as described by Karlsson^23^ and Zetterberg and Bendlin.^24^

Statistical tests of the IVIM parameters were derived through multiple regression in SPM12 under Matlab environment (R2023a), in which the IVIM parameters were used as dependent variables, and age, sex, *APOE* status, amyloid-PET positivity, and the continuous measures of AD, neurodegeneration, and neuroinflammation biomarkers were considered separately as independent variables. Age, sex, and *APOE*-ε4 were used as confounding variables unless they were the variables of interest. Clusters smaller than 50 voxels were excluded (k ≥ 50). We examined for both positive and negative associations at a significance level of 0.001 (uncorrected for multiple comparisons), followed by false discovery rate (FDR) correction at the cluster level (*P* < 0.05). For the analysis of GM volume maps, voxel-wise multiple regression was conducted using an in-house Matlab script. This script generated *t*-stat maps for each IVIM parameter, which were subsequently processed in SPM12 for correction of multiple comparisons.

Finally, we investigated the association between GM volume and water diffusion in regions showing amyloid-related increases in GM volume. These regions were identified through multiple regression in two separate analyses: (a) using amyloid-PET positivity, and (b) using CSF Aβ42/40 as the dependent variable. The ratio of CSF Aβ42 measured with Elecsys® to CSF Aβ40 measured with the NeuroToolKit was utilized to identify amyloid-related GM changes to align more closely with the analysis in the study by Salvadó *et al*..^7^ Age, sex, and total intracranial volume (TIV) were used as confounding variables (*P* < 0.005 uncorrected; k ≥ 100). We then assessed the association between GM volume and each diffusion parameter using multiple regression models, in which each diffusion parameter was treated as the independent variable, GM volume as the dependent variable, and age, sex, and TIV as confounding variables.

## Results

### Participant characteristics

A total of 297 participants were included and divided into three groups according to their CSF amyloid-β (A) and CSF p-tau181 tau (T) levels, using cut-offs defined in Milà-Alomà *et al.*^22^ (Aβ42/40 ≤ 0.071, p-tau181 > 24 pg/ml): A − T − (*n* = 192), A + T − (*n* = 81), and A + T + (*n* = 24) (Table 1). Of these participants, 151 had available Aβ PET scans. Among them, 121 had Centiloid values below 12 CLs (amyloid-PET negative), while 30 had values above 12 CLs (amyloid-PET positive).

**Table 1:** Study participants’ demographics and characteristics of Alzheimer’s disease

	A − T − (*n* = 192)	A + T − (*n* = 81)	A + T + (*n* = 24)	A − T − versus A + T − (*P value*)	A − T − versus A + T + (*P* value)	A + T − versus A + T + (*P* value)
**Age, years**	60.1 (4.4) [50, 68]	61.2 (4.9) [50, 71]	64.0 (4.7) [52, 73]	0.073	0.001	0.016
**Females, N (%)**	128 (66.7%)	50 (61.7%)	17 (70.8%)	0.434	0.682	0.415
***APOE-*ε4 carriers, N (%)**	76 (39.6%)	67 (82.7%)	14 (58.3%)	<0.001	<0.001	<0.001
**Aβ PET, CL**	−3.9 (6.5)	12.6 (17.2)	27.9 (22.3)	<0.001	<0.001	0.029
**CSF Aβ42/Aβ40**	0.087 (0.009)	0.054 (0.011)	0.045 (0.011)	<0.001	<0.001	0.001
**CSF p-tau181, pg/ml**	14.2 (4.1)	15.2 (4.0)	30.0 (5.1)	0.079	<0.001	<0.001
**TIV-adjusted hippocampal volume, mm^3^**	7617.0 (555.6)	7690.8 (529.8)	7501.4 (766.4)	0.320	0.509	0.298
**MMSE, score**	29.1 (0.9) [27, 30]	29.3 (0.9) [27, 30]	28.8 (1.2) [27, 30]	0.213	0.199	0.076
**PACC, *z*-score**	0.0 (0.7)	0.1 (0.7)	−0.4 (0.7)	0.142	0.023	0.004

Data are expressed as mean (SD) for all measurements, and number (percentage) for the variables ‘females’ and ‘*APOE*-ε4 carriers’. The ranges for age and MMSE score are indicated in square brackets. The *P* values were computed with a *t*-test for all the measurements and *χ*^2^ test for the variables ‘females’ and ‘*APOE*-ε4 carriers’.

Aβ: amyloid-beta; A−: normal levels of Aβ proteins; A+: altered levels of Aβ proteins; *APOE*-ε4: apolipoprotein E epsilon 4; CL: centiloid; MMSE: Mini-Mental State Examination; PACC: Preclinical Alzheimer Cognitive Composite; PET: positron emission tomography; T−: normal levels of tau proteins; T+: accumulation of tau proteins; TIV: total intracranial volume.

The mean age was higher in the A + T + group (64.0 years), compared with the A − T − (60.1 years, *P* = 0.001) and A + T − (61.2 years, *P* = 0.016) groups. The majority of the participants were females (*n* = 195, 65.7%), with no significant differences between groups (Table 1).

The remaining description of the participants’ characteristics is in the Supplemental Material and Table 1.

### Association of the IVIM parameters with participant characteristics in the control group

IVIM parametric maps averaged across subjects within the A − T − control group showed that, in the GM, slow DC and perfusion fraction values were higher than in other regions; and, in the WM, fast DC and SSP values were higher than in other regions (Supplementary Fig. 1).

**Figure 2:**
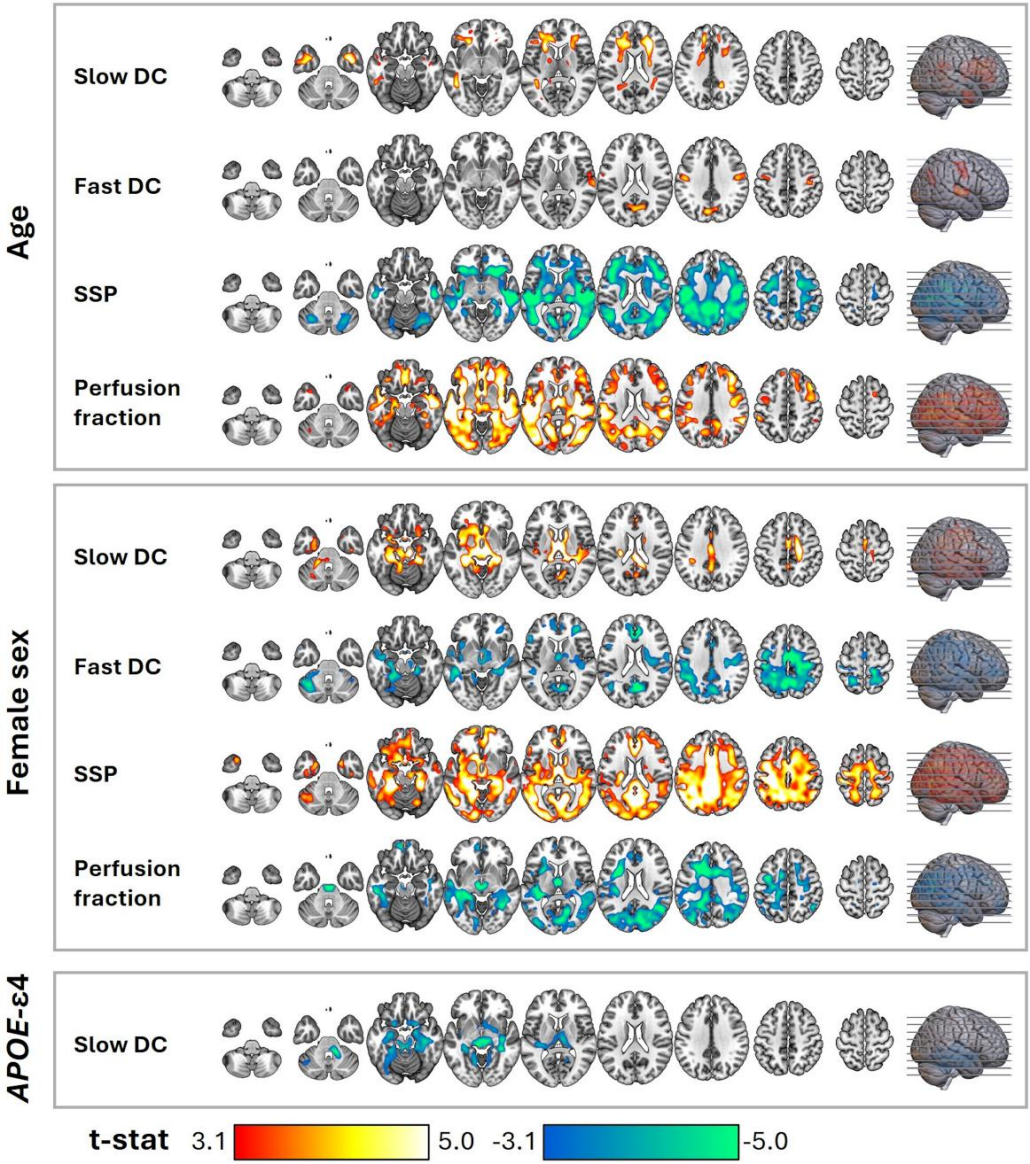
**Association of the IVIM parameters with demographic variables and the genetic factor *APOE*-ε4, in the A − T − control group (*n* = 192).** Regions with significant associations were identified through multiple regression considering each IVIM parameter as a dependent variable and age, female sex, and *APOE-ε4* as covariates. The statistical maps present *t*-stats thresholded at a voxel-level significance of *P* < 0.001 and corrected for multiple comparisons at the cluster level using FDR (*P* < 0.05), excluding clusters smaller than 50 voxels. Warm and cold colours indicate positive and negative associations, respectively. The axial slices presented across the columns (left-right) are indicated by lines on the brain surface in the rightmost column (bottom-up). *APOE*-ε4: apolipoprotein E epsilon 4; DC: diffusion coefficient; IVIM: intravoxel incoherent motion; SSP: slow signal portion.

Different participant characteristics correlated with the IVIM parameters in the A − T − group (Fig. 2). Age and female sex presented a strong positive association with slow DC. Female sex also showed a strong positive association with SSP, whereas age showed a strong negative association with it. Moreover, age had a strong positive association with perfusion fraction, whereas female sex had a strong negative association with it. Finally, age was positively associated with fast DC, whereas female sex was negatively associated with it. These associations were mainly in the GM of the cingulum. *APOE*-ε4 was negatively associated with slow DC in the basal ganglia. Given these observed effects, we used age, sex, and *APOE*-ε4 as confounding variables in our subsequent analyses that included all the subjects of the study.

### Association of the IVIM parameters with GM volume, AD and neurodegeneration biomarkers

**Figure 3:**
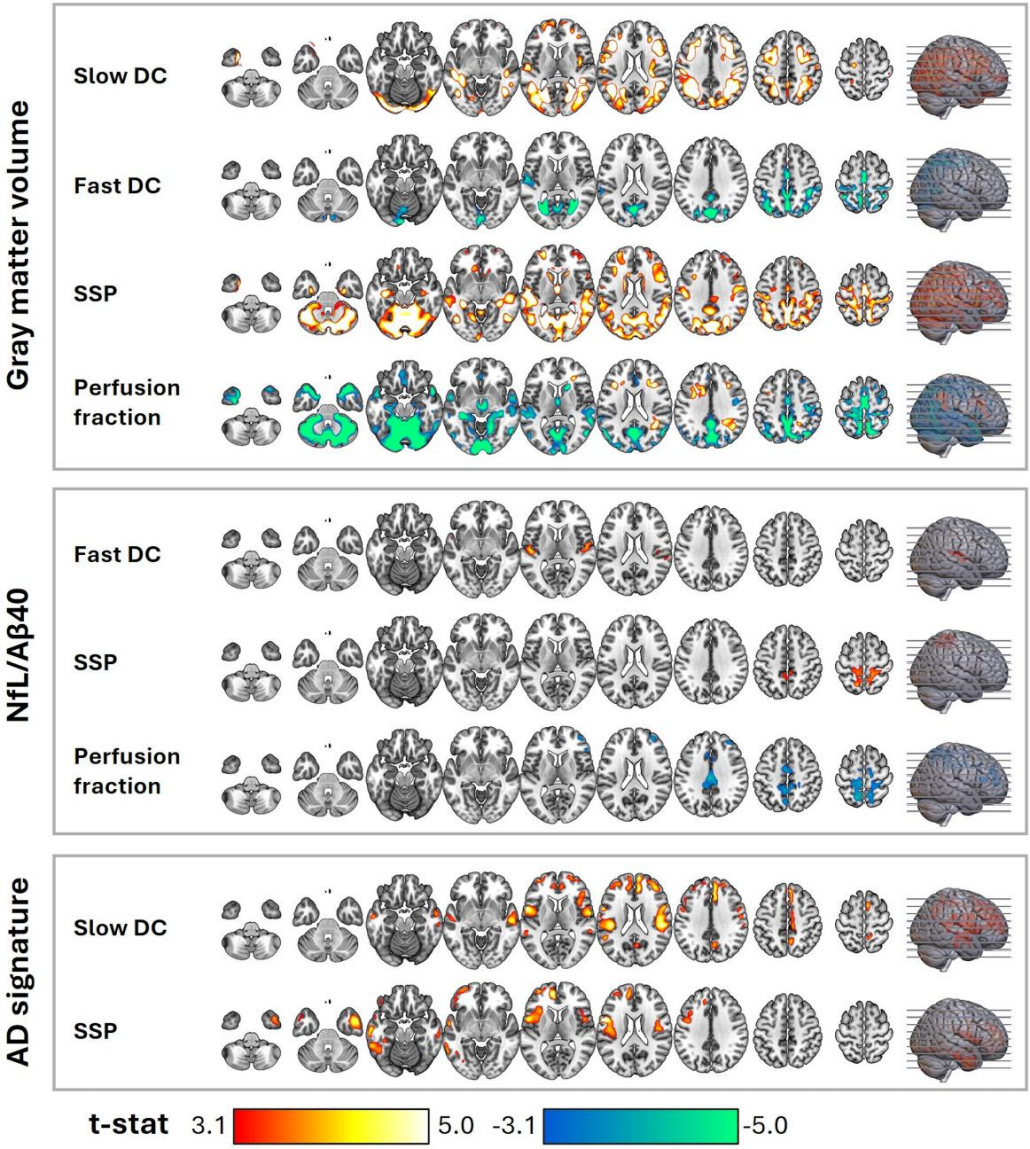
**Association of the IVIM parameters with GM volume and neurodegeneration biomarkers in all subjects (*n* = 297).** Regions with significant associations were identified through multiple regression considering each IVIM parameter as a dependent variable, biomarkers as covariates, and age, sex, and *APOE*-ε4 as confounding variables. The statistical maps present *t*-stats thresholded at a voxel-level significance of *P* < 0.001 and corrected for multiple comparisons at the cluster level using FDR (*P* < 0.05), excluding clusters smaller than 50 voxels. Warm and cold colours indicate positive and negative associations, respectively. The axial slices presented across the columns (left-right) are indicated by lines on the brain surface in the rightmost column (bottom-up). Aβ40: amyloid beta 40; AD: Alzheimer’s disease; APOE-ε4: apolipoprotein E epsilon 4; DC: diffusion coefficient; GM: gray matter; IVIM: intravoxel incoherent motion; NfL: neurofilament light; SSP: slow signal portion.

Voxel-wise analyses revealed distinct associations between GM volume and IVIM parameters (Fig. 3). GM volume was positively associated with slow DC in frontal and temporal cortices, as well as in medial occipital and subcortical regions. SSP also showed positive associations in posterior parietal areas, medial temporal structures, and the cingulate cortex. In contrast, GM volume was negatively associated with fast DC in posterior parietal and occipital regions. Perfusion fraction exhibited a mixed pattern, showing negative associations in medial parietal areas and the cerebellum, and positive associations, though limited to smaller areas, in frontal and lateral temporal cortices.

We did not find significant differences in IVIM parameters when comparing the A + T − and A + T + groups to the A − T − control group. Similarly, in the 151 participants with available amyloid-PET Centiloid values, no IVIM parameter showed statistically significant associations with amyloid-PET status. In addition, we did not find any associations of the levels of CSF Aβ42/Aβ40 and p-tau181/Aβ40 with the IVIM parameters when pooling data from all three groups (A − T−, A + T−, and A + T+).

In contrast, the biomarkers of neurodegeneration were associated with several IVIM parameters in different brain regions (Fig. 3). CSF NfL/Aβ40 presented a positive association with fast DC in the posterior insula, and with SSP in the precuneus and postcentral gyrus. Furthermore, it presented a negative association with perfusion fraction in the cingulate gyrus, precuneus, and superior parietal lobe.

The AD signature was positively associated with slow DC in the medial frontal gyrus, insula, inferior frontal gyrus, superior temporal gyrus, and precentral gyrus; and with SSP in the medial frontal gyrus, insula, precentral gyrus, medial temporal gyrus, and fusiform gyrus. The hippocampal volume did not show any associations with any of the IVIM parameters.

### Association between the IVIM parameters and neuroinflammation biomarkers

**Figure 4:**
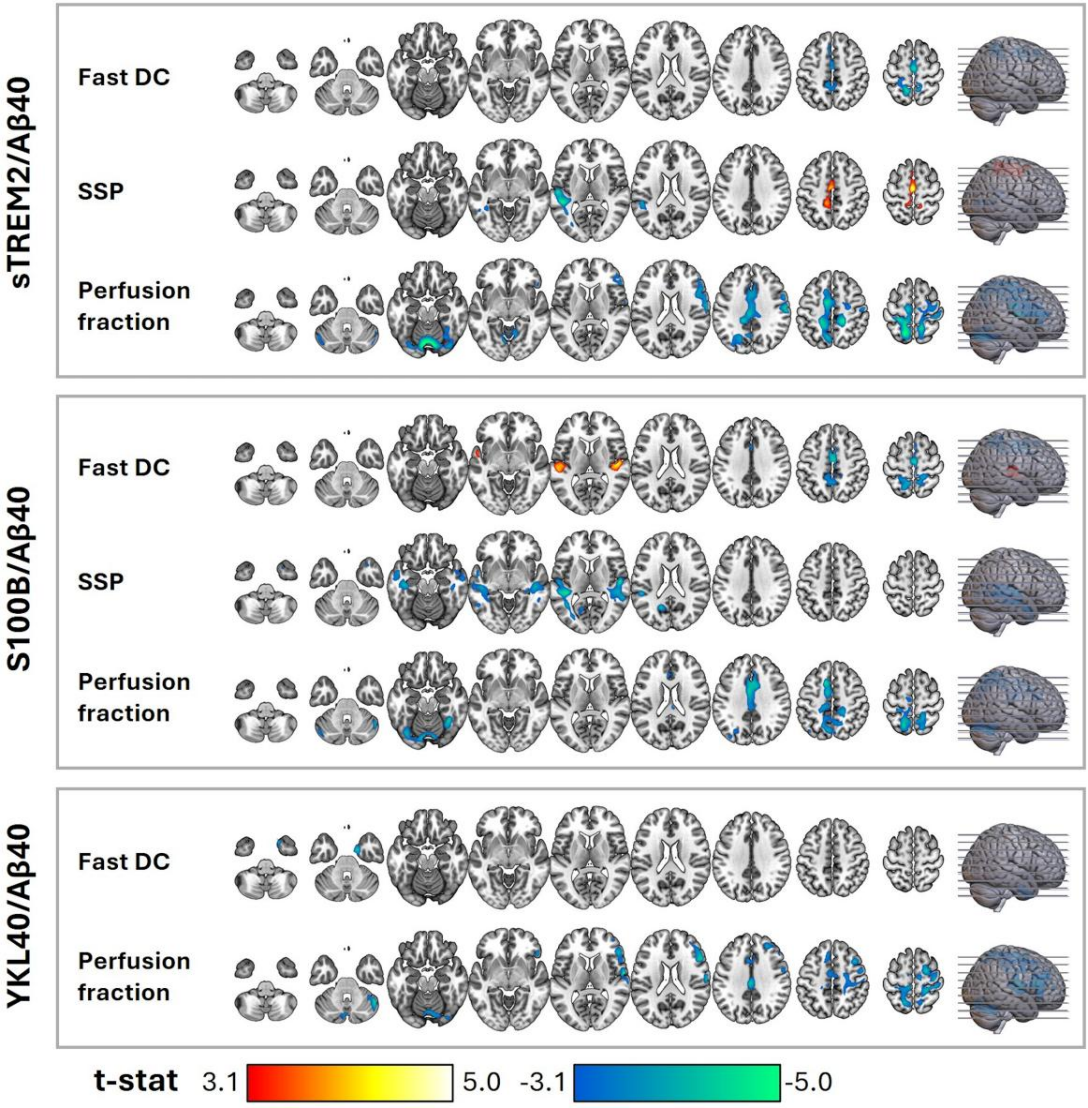
**Association between the IVIM parameters and neuroinflammation biomarkers in all subjects (*n* = 297)—part 1.** Regions with significant associations were identified through multiple regression, considering each IVIM parameter as a dependent variable, biomarkers as covariates, and age, sex, and *APOE*-ε4 as confounding variables. The statistical maps present *t*-stats thresholded at a voxel-level significance of *P* < 0.001 and corrected for multiple comparisons at the cluster level using FDR (*P* < 0.05), excluding clusters smaller than 50 voxels. Warm and cold colours indicate positive and negative associations, respectively. The axial slices presented across the columns (left-right) are indicated by lines on the brain surface in the rightmost column (bottom-up). Aβ40: amyloid beta 40; *APOE*-ε4: apolipoprotein E epsilon 4; DC: diffusion coefficient; IVIM: intravoxel incoherent motion; SSP: slow signal portion; sTREM2: soluble triggering receptor expressed on myeloid cells 2; S100B: S100 calcium-binding protein; YKL-40: chitinase-3-like protein 1.

**Figure 5:**
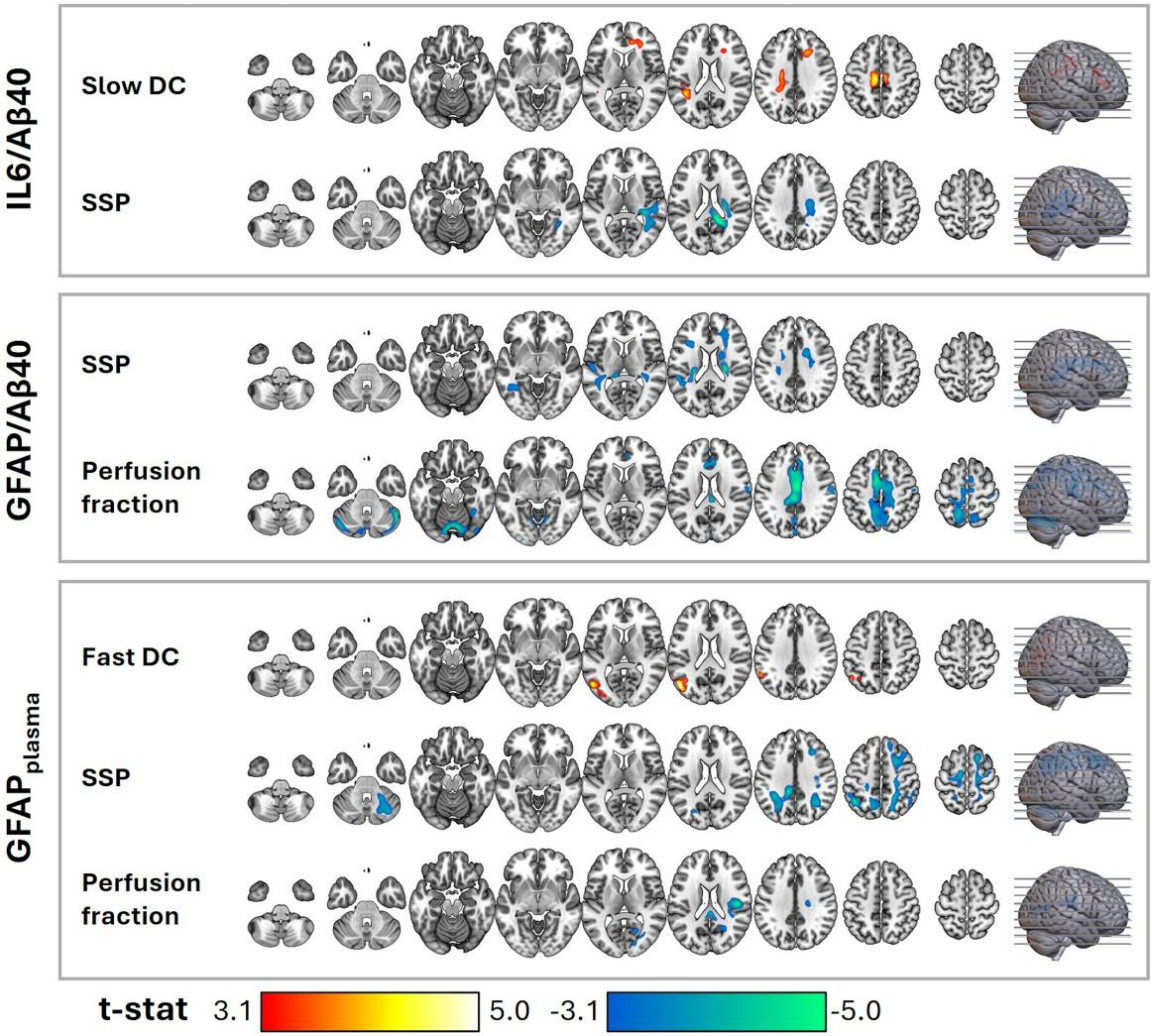
**Association between the IVIM parameters and neuroinflammation biomarkers in all subjects (*n* = 297)—part 2.** Regions with significant associations were identified through multiple regression, considering each IVIM parameter as a dependent variable, biomarkers as covariates, and age, sex, and *APOE*-ε4 as confounding variables. The statistical maps present *t*-stats thresholded at a voxel-level significance of *P* < 0.001 and corrected for multiple comparisons at the cluster level using FDR (*P* < 0.05), excluding clusters smaller than 50 voxels. Warm and cold colours indicate positive and negative associations, respectively. The axial slices presented across the columns (left-right) are indicated by lines on the brain surface in the rightmost column (bottom-up). Aβ40: amyloid beta 40; *APOE*-ε4: apolipoprotein E epsilon 4; DC: diffusion coefficient; GFAP: glial fibrillary acidic protein; IL-6: interleukin-6; IVIM: intravoxel incoherent motion; SSP: slow signal portion.

The CSF biomarkers of neuroinflammation were associated with several IVIM parameters in both GM and WM (Figs 4 and 5). sTREM2/Aβ40 was negatively associated with fast DC in the cingulate gyrus and positively associated with SSP in the same region (Fig. 4). Additionally, it showed a negative association with SSP in the superior temporal gyrus. Perfusion fraction was negatively associated with sTREM2/Aβ40 in multiple regions, including the cingulate gyrus, right middle and inferior frontal gyrus, right postcentral gyrus, left precuneus, and cerebellar regions.

S100B/Aβ40 was positively associated with fast DC in the posterior insula and transverse temporal gyrus and negatively associated with fast DC in the cingulate gyrus (Fig. 4). Additionally, it showed a negative association with SSP in the transverse temporal and superior temporal gyri. Perfusion fraction was negatively associated with S100B/Aβ40 in the cingulate gyrus, precuneus, and cerebellar regions.

YKL-40/Aβ40 was negatively associated with fast DC in the uncus (Fig. 4). Additionally, it showed a negative association with perfusion fraction in the mid frontal gyrus, paracentral lobule, postcentral gyrus, precuneus, and cingulate gyrus.

IL-6/Aβ40 was positively associated with slow DC in the right frontal WM adjacent to the anterior cingulate cortex, and in the left medial frontoparietal WM (Fig. 5). Further, it showed a negative association with SSP in the right medial frontoparietal WM, as well as in the periventricular WM between the posterior insula and caudate.

GFAP/Aβ40 was negatively associated with SSP in the periventricular white matter, and with perfusion fraction in the cingulate gyrus and cerebellar regions (Fig. 5). Plasma GFAP was positively associated with fast DC in the inferior parietal lobe, supramarginal gyrus, and mid occipital and temporal gyri (Fig. 5). Further, it showed a negative association with SSP in the inferior parietal lobe, in the right mid frontal gyrus, right postcentral gyrus, and the left angular gyrus. Finally, plasma GFAP was negatively associated with perfusion fraction in the right posterior cingulate cortex and right posterior insula.

### Association between the IVIM and MD parameter maps

We also studied the correlation between the MD map and the four IVIM parameter maps in all subjects and observed that the MD map negatively correlated with slow DC, fast DC, and SSP maps, whereas it positively correlated with the perfusion fraction map (*P* < 0.001) (Supplementary Fig. 2).

### Association of GM volume with amyloid-PET positivity, CSF Aβ42/40, IVIM parameters, and MD

**Figure 6:**
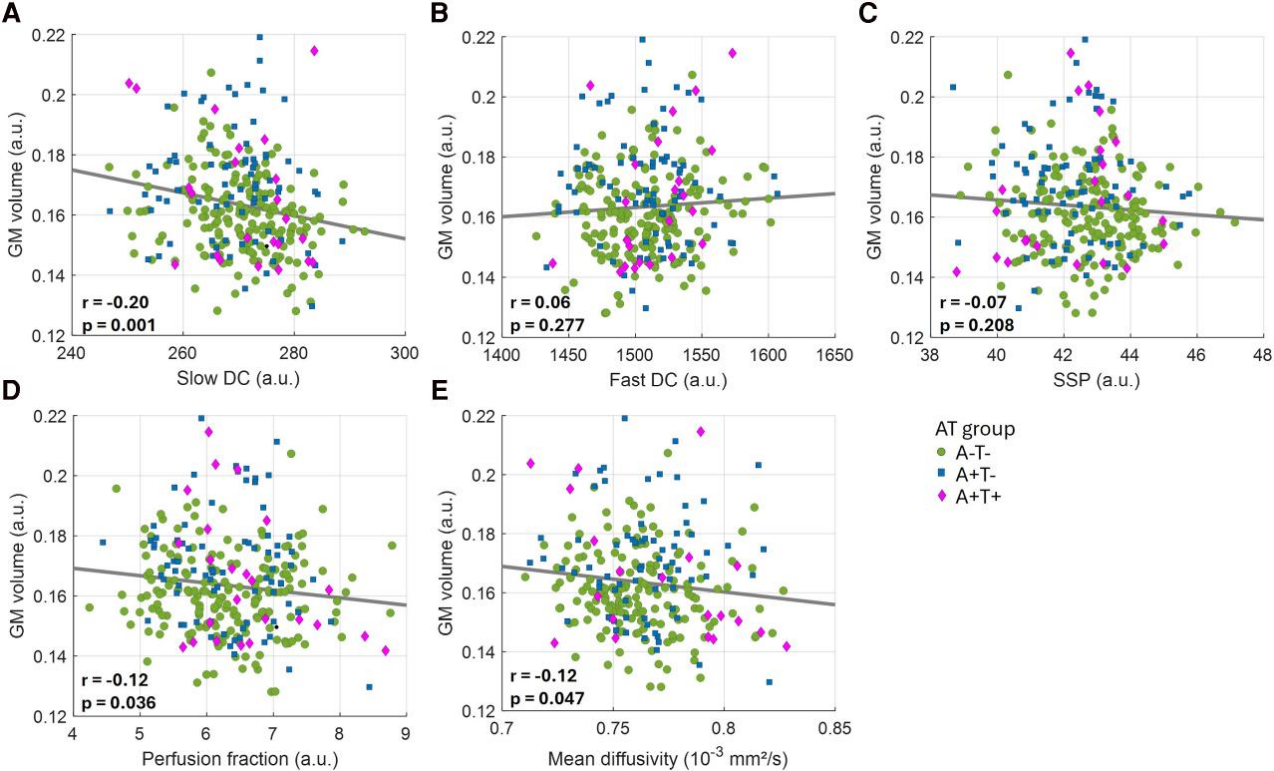
**Association between GM volume averaged across regions where GM volume was positively associated with amyloid-PET positivity and the diffusion parameters in all subjects (*n* = 297).** Significant associations between GM volume and the diffusion parameters derived from the IVIM model, namely (**A**) slow DC, (**B**) fast DC, (**C**) SSP, (**D**) perfusion fraction, as well as (**E**) the MD diffusion parameter, were identified through multiple regression, considering each diffusion parameter as the independent variable, GM volume as the dependent variable, and age, sex, and TIV as confounding variables. Each data point represents a single participant. Interaction terms between diffusion parameters and AT stage were also tested in the regression model, but were not statistically significant. a.u.: arbitrary units; DC: diffusion coefficient; GM: gray matter; intravoxel incoherent motion; MD: mean diffusivity; SSP: slow signal portion.

GM volume positively correlated with amyloid-PET positivity in the parahippocampus, fusiform gyrus, isthmus of the cingulate gyrus, paracentral lobule, and right occipital lobe (Supplementary Fig. 3). Moreover, the average GM volume across regions associated with amyloid-PET positivity showed a negative correlation with slow DC (*P* = 0.001), perfusion fraction (*P* = 0.036), and MD (*P* = 0.047) (Fig. 6). GM volume was also negatively associated with CSF Aβ42/40 levels in the parahippocampus, fusiform gyrus, isthmus of the cingulate gyrus and right occipital lobe, and rostral anterior cingulate and precuneus (Supplementary Fig. 4). Further, the average GM volume across regions associated with reduced CSF Aβ42/40 showed negative correlations with slow DC (*P* = 0.007), perfusion fraction (*P* = 0.027), and MD (*P* < 0.001) (Supplementary Fig. 5).

## Discussion

In this study, we derived a three-compartment IVIM model with four parameters to measure distinct pools of cerebral water diffusion in subjects with preclinical AD (amyloid-positive cognitively unimpaired individuals) and healthy individuals. Our results show that, in brain areas showing amyloid accumulation, the observed higher GM volumes were associated with lower slow DC, a marker of intracellular water diffusion. This finding suggests a potential link between amyloid accumulation and altered microstructural properties indicative of intracellular water retention or cell swelling. Such changes may reflect neuroinflammatory processes, possibly driven by astroglial reactivity, although further studies are needed to directly assess this relationship. These results warrant the use of IVIM parameters to study the aetiology of the brain volume reductions associated with amyloid-clearance in anti-amyloid immunotherapies, showing a clinical benefit for the participants.

Cerebral accumulation of amyloid beta in the early preclinical stages of AD is associated with a GM volume increase in brain regions that typically undergo neurodegeneration and volume decrease in later symptomatic stages; however, the underlying mechanisms remain controversial.^25^ Understanding these mechanisms could shed light on why clinical trials with anti-amyloid drugs with beneficial clinical effects have found a decrease in brain volume in early symptomatic AD.^9,10^ The use of multi-compartment models for studying cerebral water diffusion in AD has been limited,^26,27^ despite their success in distinguishing between different water pools in individuals with various neurological issues^28-31^ Particularly, three-compartment IVIM models^32,33^ are believed to better account for the existence of distinct water pools in biological tissues compared to two-compartment models. They have been shown to provide better fit to the data across various organs, including the brain,^34^ offering richer information and potentially more accurate measures of diffusion-related parameters. Considering this, we developed a three-compartment IVIM model with four parameters to measure cerebral water diffusion in subjects with preclinical AD and healthy individuals. Specifically, we derived two parameters reflecting the water diffusion rate in the intracellular and extracellular spaces, and two additional parameters representing the relative signal contributions from the intracellular water and the microvasculature.

Firstly, we characterized the IVIM parameters against demographic factors in the healthy control group. We found that age and sex were associated with all the IVIM parameters, while *APOE* status was associated only with slow DC, underscoring the importance of accounting for these factors as confounding variables in the subsequent statistical analyses. Notably, we observed an association of age with an increase in perfusion fraction in the cortical region, which aligns with findings reported by Vieni and colleagues (2020).^35^ Moreover, we observed widespread positive associations of female sex with SSP and a negative association with perfusion fraction. These patterns were spatially similar to those observed for age, albeit with reversed polarity. Importantly, sex effects were independent of age, as confirmed by matched age distributions and statistical adjustment. Our findings are consistent with previous studies showing that MD increases with age and is higher in males than in females.^36^ Note, in our data, MD was positively correlated with perfusion fraction and negatively with SSP, reinforcing the interpretation that MD may reflect mainly extracellular and vascular diffusivity. The increased SSP and decreased perfusion fraction in females may reflect greater cell density in grey matter, supporting the notion that sex-related differences in cellular composition influence water diffusion characteristics captured by IVIM parameters.

Furthermore, in the full cohort analysis, we observed robust regional associations between GM volume and the four IVIM parameters. These associations mirrored the trends previously identified in females, namely, positive correlations with slow DC and SSP, and negative correlations with fast DC and perfusion fraction, though the spatial distribution of these effects was not entirely overlapping. One potential explanation is that IVIM quantification may be influenced by CSF contamination at the voxel level, which can reduce the contribution of the GM signal. Alternatively, these patterns may reflect individual variability in regional GM volume beyond sex-related differences. Individuals with greater GM volume may exhibit a higher proportion of diffusion signal originating from intracellular water, characterized by elevated diffusion rates within these compartments.

We also identified connections between biomarkers of neurodegeneration and the IVIM parameters in cognitively unimpaired subjects, either with preclinical AD or healthy. In particular, NfL/Aβ40 and AD signature were associated with SSP in various GM regions, including frontal, parietal, and temporal areas. This supports previous research on the association of plasma NfL^37^ and cortical thickness^38^ with water diffusion in the parietal and temporal regions. However, we did not observe any associations between hippocampal volume and IVIM parameters, which contrasts with recent studies reporting links between hippocampal volume and the intermediate component fraction $f_{int}$, a diffusion parameter thought to reflect the volume fraction of the interstitial fluid compartment.^32^

Moreover, we observed an association between fluid biomarkers of neuroinflammation related to AD and our IVIM parameters. Similarly, Spotorno *et al.*^37^ found a positive association between two of the fluid biomarkers of inflammation that we analysed (CSF YKL-40 and plasma GFAP) and water diffusion. Additionally, another study found that two of the CSF biomarkers of neuroinflammation that we examined (YKL-40 and sTREM2) can predict cerebral volumetric changes over time in cognitively unimpaired individuals.^6^ Taken together, our findings and these previous studies suggest that neuroinflammation is associated with cerebral water diffusion and brain volumetric changes.

Building on this, we identified several recurring patterns across neuroinflammatory biomarkers in relation to IVIM parameters. Most notably, perfusion fraction and SSP showed consistent negative associations, although these effects did not always occur in the same regions. The widespread negative association of perfusion fraction suggests that neuroinflammation may reduce microvascular water contribution, potentially reflecting vascular compression or impaired perfusion. In parallel, reductions in SSP with astrocytic markers (e.g. CSF S100B/Aβ40, CSF GFAP/Aβ40, plasma GFAP) indicate a lower intracellular water signal when astrocytic activity is high, possibly due to increased extracellular space or altered cellular morphology.

Regarding fast DC, we observed negative associations in the cingulate cortex for both sTREM2/Aβ40 and S100B/Aβ40. Interestingly, these two biomarkers also showed negative associations with perfusion fraction in the same regions. This pattern suggests that both astrocytic and microglial activation are linked not only to impaired perfusion but also to decreased extracellular diffusion rates, which may reflect increased complexity or crowding in the extracellular space. Finally, while the overall direction of associations was largely consistent across biomarkers, spatial patterns varied. For example, SSP reductions were observed in GM for CSF sTREM2/Aβ40 and CSF S100B/Aβ40, whereas for CSF IL6/Aβ40, CSF GFAP/Aβ40, and plasma GFAP, decreases were primarily localized to GM. Further studies are needed to clarify the biological mechanisms underlying these spatial differences.

Importantly, we did not observe significant differences in IVIM parameters when comparing CSF-based AT groups (A − T − versus A + T − and A + T+) or amyloid-PET status. We also conducted post-hoc subgroup analyses to examine associations between IVIM parameters and CSF Aβ42/40 within each AT group separately; however, no significant associations were identified. Similarly, no associations were found between IVIM parameters and continuous measures of CSF Aβ42/40 or p-tau181/Aβ40, suggesting that IVIM metrics may be more sensitive to neuroinflammatory processes than to amyloid or tau pathology in cognitively unimpaired individuals. These findings contrast with those of Montal *et al*. (2018), who reported a biphasic trajectory of MD changes along the AD continuum, with decreased MD in early preclinical stages associated with amyloid deposition, and increased MD in later stages reflecting neurodegeneration. The lack of IVIM sensitivity to amyloid in our cohort may be due to differences in AT staging methodology or cohort characteristics between the two studies.

Interestingly, our voxel-wise analysis revealed widespread positive associations between GM volume and slow DC, suggesting that higher GM volume is generally linked to increased intracellular water diffusivity. This pattern may reflect healthier tissue microstructure or reduced restriction within the intracellular compartment. However, when focusing specifically on regions where GM volume was associated with amyloid-PET positivity, we observed a negative association between GM volume and slow DC. Notably, SSP, reflecting the relative contribution of intracellular water to the diffusion signal, also showed a negative association with GM volume in these regions, albeit not statistically significant. These concurrent reductions in slow DC and SSP suggest that these changes are unlikely to reflect intracellular water accumulation. Instead, they may be driven by mechanisms such as increased intracellular complexity, including glial and cytoskeletal remodelling or organelle crowding, that restrict water mobility and reduce the signal contribution from the intracellular compartment.

Given previous findings linking markers of astroglial activity (e.g. CSF YKL-40 and plasma GFAP) to both cerebral water diffusion and volumetric changes,^6,37^ our results suggest that neuroinflammation may contribute to microstructural alterations underlying GM volume increases. These changes may involve increased cellular complexity or glial remodelling that restricts water mobility. This hypothesis is particularly relevant considering that brain volume reductions are often observed following anti-amyloid therapies, which also lead to decreases in plasma GFAP levels.^9,39^ Although the precise mechanisms remain to be elucidated, these findings support the use of multi-compartment diffusion imaging to investigate the mechanisms underlying the volumetric decreases observed after clinically beneficial amyloid removal.

Building on these findings, we observed that slow DC demonstrated a stronger association with GM volume than MD, highlighting the added sensitivity of multi-compartment diffusion imaging to microstructural changes. While MD captures average diffusivity across both intra- and extracellular compartments, slow DC specifically reflects intracellular water mobility. This distinction is critical in the context of preclinical AD, where subtle intracellular changes may precede apparent neurodegeneration. By disentangling distinct diffusion components, multi-compartment models offer enhanced specificity and may outperform conventional DWI metrics in detecting early pathological processes.

Our study has some limitations. First, as a cross-sectional study, we did not investigate if DWI can track changes in water pools over time^14^; however, this study paves the way for the use of multi-compartmental DWI to better understand the effects of anti-amyloid drugs on brain structure. Second, our cohort, composed of healthy individuals and subjects in the early pre-clinical stage of AD, may explain the absence of association of IVIM parameters with either CSF or PET amyloid biomarkers. Further studies at later stages of the AD *continuum* are required to uncover the true extent of these associations. Third, given the exploratory nature of the study and limited statistical power in terms of sample size, we did not apply a correction for multiple comparisons across biomarker tests, which may increase the risk of false-positive findings. Therefore, associations reported between IVIM parameters and fluid biomarkers should be interpreted with caution. Future studies with larger sample sizes are needed to apply more stringent correction methods and validate these findings.

Additionally, we did not apply denoising or eddy-current correction to the multi-shell data due to the very low number of diffusion directions, which makes standard correction methods unsuitable or potentially unstable. Advanced denoising techniques such as MPPCA^40^ require high directional redundancy, which our protocol does not provide. Furthermore, susceptibility distortion correction was not performed due to the absence of reversed phase-encoding images. This limitation may particularly affect IVIM estimates in regions near air–tissue interfaces, such as the orbitofrontal and prefrontal cortices, where magnetic susceptibility differences from nasal sinuses can cause local geometric warping. Such distortions may lead to misalignment with anatomical templates and partial volume effects. While affine registration mitigates global misalignment, it cannot fully correct these local distortions. Future work should incorporate optimized acquisition strategies, including reversed phase-encoding images, to minimize potential bias in IVIM parameter estimation.

Lastly, our modelling approach was based on a three-compartment IVIM framework, which, while clinically feasible, does not fully adhere to current principles of biophysical modelling of diffusion MRI. Advanced models leveraging multidimensional diffusion-relaxation encoding (Lampinen *et al*., 2023; Novikov *et al*., 2019) can provide richer microstructural information and reduce parameter bias. Future studies should explore these approaches to validate and extend our findings.

Despite these limitations, our study has various strengths. We examined a cohort much larger than those in previous studies,^26,32^ which was well-characterised with a range of fluid and MRI biomarkers. Moreover, we developed and utilized a new three-compartment DWI model that distinguishes between intracellular and extracellular water diffusion, providing a more accurate characterisation of cerebral water pools.

Multi-compartment diffusion-weighted imaging enables the measurement of cerebral water IVIM parameters associated with CSF biomarkers of neuroinflammation and cerebral volume alterations. This method may help disentangle the aetiology of cerebral volume reductions in individuals receiving anti-amyloid therapies in early symptomatic AD. Further studies are needed to better understand whether multi-compartment DWI can distinguish between different stages of AD and other neurodegenerative diseases.

## Supplementary Material

fcag075_Supplementary_Data

## Data Availability

The data supporting the findings of this study are available on request from the corresponding author. The data are not publicly available due to privacy or ethical restrictions. Code used for the analyses is available at the following GitHub repository: https://github.com/mkassinopoulos/2026_multib_paper_AlfaPLUS.

## Appendix 1

Collaborators of the ALFA study are: Müge Akinci, Federica Anastasi, Annabella Beteta, Raffaele Cacciaglia, Lidia Canals, Alba Cañas, Carme Deulofeu, Maria Emilio, Irene Cumplido-Mayoral, Marta del Campo, Carme Deulofeu, Ruth Dominguez, Maria Emilio, Sherezade Fuentes, Marina García, Laura Hernández, Gema Huesa, Laura Iglesias, Esther Jiménez, David López-Martos, Paula Marne, Tania Menchón, Paula Ortiz-Romero, Eleni Palpatzis, Wiesje Pelkmans, Albina Polo, Sandra Pradas, Mahnaz Shekari, Lluís Solsona, Anna Soteras, Núria Tort-Colet, Marc Vilanova and Natalia Vilor Tejedor.

## References

[1] Scheltens P, De Strooper B, Kivipelto M, et al Alzheimer’s disease. Lancet. 2021;397(10284):1577–1590. 33667416 10.1016/S0140-6736(20)32205-4 PMC8354300

[2] Heneka MT, Carson MJ, El Khoury J, et al Neuroinflammation in Alzheimer’s disease. Lancet Neurol. 2015;14(4):388–405. 25792098 10.1016/S1474-4422(15)70016-5 PMC5909703

[3] Montal V, Vilaplana E, Alcolea D, et al Cortical microstructural changes along the Alzheimer’s disease continuum. Alzheimers Dement. 2018;14(3):340–351. 29080407 10.1016/j.jalz.2017.09.013

[4] Hayek D, Ziegler G, Kleineidam L, et al Different inflammatory signatures based on CSF biomarkers relate to preserved or diminished brain structure and cognition. Mol Psychiatry. 2024;29:992–1004. 38216727 10.1038/s41380-023-02387-3 PMC11176056

[5] Gispert JD, Monté GC, Falcon C, et al CSF YKL-40 and pTau181 are related to different cerebral morphometric patterns in early AD. Neurobiol Aging. 2016;38:47–55. 26827642 10.1016/j.neurobiolaging.2015.10.022

[6] Falcon C, Monté-Rubio GC, Grau-Rivera O, et al CSF glial biomarkers YKL40 and sTREM2 are associated with longitudinal volume and diffusivity changes in cognitively unimpaired individuals. Neuroimage Clin. 2019;23:101801. 30978656 10.1016/j.nicl.2019.101801 PMC6458453

[7] Salvadó G, Shekari M, Falcon C, et al Brain alterations in the early Alzheimer’s continuum with amyloid-β, tau, glial and neurodegeneration CSF markers. Brain Commun. 2022;4(3):fcac134. 35702732 10.1093/braincomms/fcac134 PMC9185381

[8] Gispert JD, Suárez-Calvet M, Monté GC, et al Cerebrospinal fluid sTREM2 levels are associated with gray matter volume increases and reduced diffusivity in early Alzheimer’s disease. Alzheimers Dement. 2016;12(12):1259–1272. 27423963 10.1016/j.jalz.2016.06.005

[9] van Dyck CH, Swanson CJ, Paul A, et al Lecanemab in early Alzheimer’s disease. N Engl J Med. 2023;388(1):9–21. 36449413 10.1056/NEJMoa2212948

[10] Sims JR, Zimmer JA, Evans CD, et al Donanemab in early symptomatic Alzheimer disease: The TRAILBLAZER-ALZ 2 randomized clinical trial. JAMA. 2023;330(6):512–527. 37459141 10.1001/jama.2023.13239 PMC10352931

[11] Alves F, Kalinowski P, Ayton S. Accelerated brain volume loss caused by anti–β-amyloid drugs. Neurology. 2023;100(20):e2114–e2124. 36973044 10.1212/WNL.0000000000207156 PMC10186239

[12] Fox NC, Black RS, Gilman S, et al Effects of Aβ immunization (AN1792) on MRI measures of cerebral volume in Alzheimer disease. Neurology. 2005;64(9):1563–1572. 15883317 10.1212/01.WNL.0000159743.08996.99

[13] Belder CRS, Boche D, Nicoll JAR, et al Brain volume change following anti-amyloid β immunotherapy for Alzheimer’s disease: Amyloid-removal-related pseudo-atrophy. Lancet Neurol. 2024;23(10):1025–1034. 39304242 10.1016/S1474-4422(24)00335-1

[14] van Baalen S, Leemans A, Dik P, Lilien MR, ten Haken B, Froeling M. Intravoxel incoherent motion modeling in the kidneys: Comparison of mono-, bi-, and triexponential fit. J Magn Reson Imaging. 2017;46(1):228–239. 27787931 10.1002/jmri.25519 PMC5484284

[15] Jack Jr CR, Wiste HJ, Weigand SD, et al Defining imaging biomarker cut points for brain aging and Alzheimer’s disease. Alzheimers Dement. 2017;13(3):205–216. 27697430 10.1016/j.jalz.2016.08.005 PMC5344738

[16] Schwarz CG, Gunter JL, Wiste HJ, et al A large-scale comparison of cortical thickness and volume methods for measuring Alzheimer’s disease severity. Neuroimage Clin. 2016;11:802–812. 28050342 10.1016/j.nicl.2016.05.017 PMC5187496

[17] Molinuevo JL, Gramunt N, Gispert JD, et al The ALFA project: A research platform to identify early pathophysiological features of Alzheimer’s disease. Alzheimers Dement. 2016;2(2):82–92. 10.1016/j.trci.2016.02.003 PMC5644283 29067295

[18] Donohue MC, Sperling RA, Salmon DP, et al The preclinical Alzheimer cognitive composite: Measuring amyloid-related decline. JAMA Neurol. 2014;71(8):961–970. 24886908 10.1001/jamaneurol.2014.803 PMC4439182

[19] Sánchez-Benavides G, Suárez-Calvet M, Milà-Alomà M, et al Amyloid-β positive individuals with subjective cognitive decline present increased CSF neurofilament light levels that relate to lower hippocampal volume. Neurobiol Aging. 2021;104:24–31. 33962331 10.1016/j.neurobiolaging.2021.02.026

[20] Conklin J, Heyn C, Roux M, Cerny M, Wintermark M, Federau C. A simplified model for intravoxel incoherent motion perfusion imaging of the brain. AJNR Am J Neuroradiol. 2016;37(12):2251–2257. 27561834 10.3174/ajnr.A4929 PMC7963855

[21] Jenkinson M, Beckmann CF, Behrens TEJ, Woolrich MW, Smith SM. FSL. Neuroimage. 2012;62(2):782–790. 21979382 10.1016/j.neuroimage.2011.09.015

[22] Milà-Alomà M, Salvadó G, Gispert JD, et al Amyloid beta, tau, synaptic, neurodegeneration, and glial biomarkers in the preclinical stage of the Alzheimer’s continuum. Alzheimers Dement. 2020;16(10):1358–1371. 32573951 10.1002/alz.12131 PMC7586814

[23] Karlsson L, Vogel J, Arvidsson I, et al Cerebrospinal fluid reference proteins increase accuracy and interpretability of biomarkers for brain diseases. Neuroscience. 2024;15:3676. 10.1038/s41467-024-47971-5 PMC11063138 38693142

[24] Zetterberg H, Bendlin BB. Biomarkers for Alzheimer’s disease—preparing for a new era of disease-modifying therapies. Mol Psychiatry. 2021;26(1):296–308. 32251378 10.1038/s41380-020-0721-9 PMC8172244

[25] Barkhof F, Knopman DS. Brain shrinkage in anti-β-amyloid Alzheimer trials: Neurodegeneration or pseudoatrophy? Neurology. 2023;100(20):941–942. 36973045 10.1212/WNL.0000000000207268

[26] Bergamino M, Nespodzany A, Baxter LC, et al Preliminary assessment of intravoxel incoherent motion diffusion-weighted MRI (IVIM-DWI) metrics in Alzheimer’s disease. J Magn Reson Imaging. 2020;52(6):1811–1826. 32621405 10.1002/jmri.27272

[27] Vogt NM, Hunt JFV, Adluru N, et al Interaction of amyloid and tau on cortical microstructure in cognitively unimpaired adults. Alzheimers Dement. 2022;18(1):65–76. 33984184 10.1002/alz.12364 PMC8589921

[28] Liu Z, Xiao X. The use of multi b values diffusion-weighted imaging in patients with acute stroke. Neuroradiology. 2013;55(3):371–376. 23334433 10.1007/s00234-012-1129-2

[29] Zhu G, Federau C, Wintermark M, et al Comparison of MRI IVIM and MR perfusion imaging in acute ischemic stroke due to large vessel occlusion. Int J Stroke. 2020;15(3):332–342. 31480940 10.1177/1747493019873515

[30] Bagnato F, Franco G, Li H, et al Probing axons using multi-compartmental diffusion in multiple sclerosis. Ann Clin Transl Neurol. 2019;6(9):1595–1605. 31407532 10.1002/acn3.50836 PMC6764633

[31] Tachibana Y, Aida N, Niwa T, et al Analysis of multiple B-value diffusion-weighted imaging in pediatric acute encephalopathy. PLoS One. 2013;8(6):e63869. 23755112 10.1371/journal.pone.0063869 PMC3670889

[32] van der Thiel MM, Freeze WM, Verheggen ICM, et al Associations of increased interstitial fluid with vascular and neurodegenerative abnormalities in a memory clinic sample. Neurobiol Aging. 2021;106:257–267. 34320463 10.1016/j.neurobiolaging.2021.06.017

[33] Xia N, Li Y, Xue Y, et al Intravoxel incoherent motion diffusion-weighted imaging in the characterization of Alzheimer’s disease. Brain Imaging Behav. 2022;16(2):617–626. 34480258 10.1007/s11682-021-00538-0

[34] Zeng Q, Shi F, Zhang J, Ling C, Dong F, Jiang B. A modified tri-exponential model for multi-b-value diffusion-weighted imaging: A method to detect the strictly diffusion-limited compartment in brain. Front Neurosci. 2018;12:102. 29535599 10.3389/fnins.2018.00102 PMC5834430

[35] Vieni C, Ades-Aron B, Conti B, et al Effect of intravoxel incoherent motion on diffusion parameters in normal brain. NeuroImage. 2020;204:116228. 31580945 10.1016/j.neuroimage.2019.116228 PMC6886883

[36] Lawrence KE, Nabulsi L, Santhalingam V, et al Age and sex effects on advanced white matter microstructure measures in 15,628 older adults: A UK biobank study. Brain Imaging Behav. 2021;15(6):2813–2823. 34537917 10.1007/s11682-021-00548-y PMC8761720

[37] Spotorno N, Strandberg O, Stomrud E, et al Diffusion MRI tracks cortical microstructural changes during the early stages of Alzheimer’s disease. Brain. 2024;147(3):961–969. 38128551 10.1093/brain/awad428 PMC10907088

[38] Weston PSJ, Poole T, Nicholas JM, et al Measuring cortical mean diffusivity to assess early microstructural cortical change in presymptomatic familial Alzheimer’s disease. Alz Res Therapy. 2020;12(1):112. 10.1186/s13195-020-00679-2 PMC7499910 32943095

[39] Pontecorvo MJ, Lu M, Burnham SC, et al Association of donanemab treatment with exploratory plasma biomarkers in early symptomatic Alzheimer disease: A secondary analysis of the TRAILBLAZER-ALZ randomized clinical trial. JAMA Neurol. 2022;79(12):1250–1259. 36251300 10.1001/jamaneurol.2022.3392 PMC9577883

[40] Veraart J, Novikov DS, Christiaens D, Ades-aron B, Sijbers J, Fieremans E. Denoising of diffusion MRI using random matrix theory. NeuroImage. 2016;142:394–406. 27523449 10.1016/j.neuroimage.2016.08.016 PMC5159209

