# Supplementary material for: Intracellular fluid accumulation underlies brain volume increases in early Alzheimer’s disease
Source: Brain Commun. 2026 Mar 10;8(2):fcag075. doi: 10.1093/braincomms/fcag075 (PMC13009408; doi:10.1093/braincomms/fcag075)
Supplement: fcag075_Supplementary_Data [file fcag075_supplementary_data.pdf]

Supplementary Material

Methods

Amyloid beta (Aβ) PET

Aβ PET scans were acquired for 151 out of the 297 participants to analyze the amyloid status, using [18F]-flutemetamol as a radiotracer. Scans were performed after a cranial computed tomography scan for attenuation correction on a Biograph CT scanner (Siemens Healthcare, Erlangen, Germany) at the Hospital Clínic, Barcelona, Spain. Images were acquired for 20 minutes (four frames of five minutes each) 90 minutes after injection (mean ± SD 90.15 ± 7.36 minutes) of an IV bolus [^{18}F]-flutemetamol dose of 185 MBq (range 104.25–218.3 MBq, mean ± SD 191.75 ± 14.04 MBq). Processing was performed following a validated Centiloid pipeline [1,2] with SPM12 [3]. Finally, we calculated Centiloid values from the mean values of the standard Global Cortical Average target region with the whole cerebellum as reference region using the previously calibrated transformation [1,2]. Amyloid-PET positivity was defined as a Centiloid value greater than 12.

Apolipoprotein E (APOE) genotyping

We obtained total DNA from cellular blood fraction by proteinase K digestion followed by alcohol precipitation. Samples were genotyped for two single nucleotide polymorphisms, rs429358 and rs7412, to define the *APOE*-ε2, ε3, and ε4 alleles [4], the latter being a well-known risk factor for Alzheimer's disease (AD) [5,6]. We classified participants as *APOE*-ε4 carriers (with one or two alleles) or non-carriers.

Fluid sampling and biomarkers analysis

Participants underwent lumbar puncture for CSF and blood sample extraction within one year of the MRI acquisition. We analyzed CSF biomarkers of AD (Aβ and phosphorylated tau proteins), neurodegeneration (neurofilament light [NfL]), and neuroinflammation (soluble triggering

receptor expressed on myeloid cells 2 [sTREM2], S100 calcium-binding protein [S100B], chitinase-3-like protein 1 [YKL-40], interleukin-6 [IL-6]), and glial fibrillary acidic protein [GFAP]), as well as plasma glial fibrillary acidic protein (GFAP), that have been identified in previous AD studies [6–9].

CSF biomarker and blood samples were obtained through standard procedures and have previously been described elsewhere [7,10]. We measured core prototype CSF biomarkers [6] using the NeuroToolKit, a panel of exploratory robust prototype assays (Aβ42 and Aβ40) and Elecsys® (Aβ42 and pTau181) on an automated Cobas® e 601 module (all Roche Diagnostics International Ltd, Rotkreuz, Switzerland). While most analyses utilized Aβ42 values obtained from the NeuroToolKit panel, Elecsys® Aβ42 were specifically employed to identify GM volume increments associated with increased amyloid load, aligning with the analysis in the study by Salvadó *et al.*[11]. The other biomarkers from CSF (NfL, sTREM2, S100B, YKL-40, IL6, and GFAP) were measured with robust prototype assays as part of the NeuroToolKit on Cobas® e 411 analyzer and e 601 module (Roche Diagnostics International Ltd, Rotkreuz, Switzerland). Plasma GFAP was determined with the Simoa HD-X (Quanterix) platform using the commercial Neurology 4-Plex E assay (N4PE) [12]. All fluid biomarkers were measured at the Clinical Neurochemistry Laboratory, Sahlgrenska University Hospital, Mölndal, Sweden.

To ensure that inter-individual differences in CSF levels do not drive differences in mean CSF protein levels, we used CSF Aβ40 as a reference protein [13]. We defined Aβ and tau positivity using pre-established cut-offs as Aβ42/40 (NeuroToolKit) ≤ 0.071, and p-tau181 > 24 pg/ml [7]. We then classified the individuals by amyloid tau (AT) groups and excluded A-T+ participants from the study as they are thought to reflect non-AD pathological changes [13,14].

Supplementary Results

Additional participant characteristics

We found significantly more *APOE*-ε4 carriers in the A+T- (82.7%) and the A+T+ groups (58.3%), compared with the control A-T- group (39.6%) ($p<0.001$); the number of *APOE*-ε4 carriers was also significantly higher in the A+T- group than in the A+T+ group ($p<0.001$) **(Table 1)**.

As expected, the level of CSF Aβ42/40 was significantly lower in the A+T+ group (0.045), compared with the A-T- (0.087, $p<0.001$) and A+T- (0.054, $p = 0.001$) groups; and in the A+T- group, compared with the A-T- ($p<0.001$). CSF p-tau181 levels were higher in the A+T+ group (30.0 pg/ml) compared with the A-T- (14.2 pg/ml, $p<0.001$) and A+T- (15.2 pg/ml, $p<0.001$) groups. Aβ PET Centiloids (CL) were significantly higher in the A+T+ group (27.9 CL), compared with the A-T- (-3.9 CL, $p<0.001$) and A+T- (12.6 CL, $p = 0.029$) groups; and in the A+T- group compared with the A-T- ($p<0.001$) **(Table 1)**.

No differences were observed between the three groups in total intracranial volume (TIV)-adjusted hippocampal volumes (A-T-, 7617.0 mm^3; A+T-, 7690.8 mm^3; and A+T+, 7501.4 mm^3) or in Mini-Mental State Examination (MMSE scores: A-T-, 29.1; A+T-, 29.3; and A+T+, 28.8). In contrast, the Preclinical Alzheimer Cognitive Composite (PACC) score was significantly lower in the A+T+ group (-0.4) compared with A-T- (0.0, $p<0.023$) and A+T- (0.1, $p<0.004$) **(Table 1)**.

Supplementary Figures

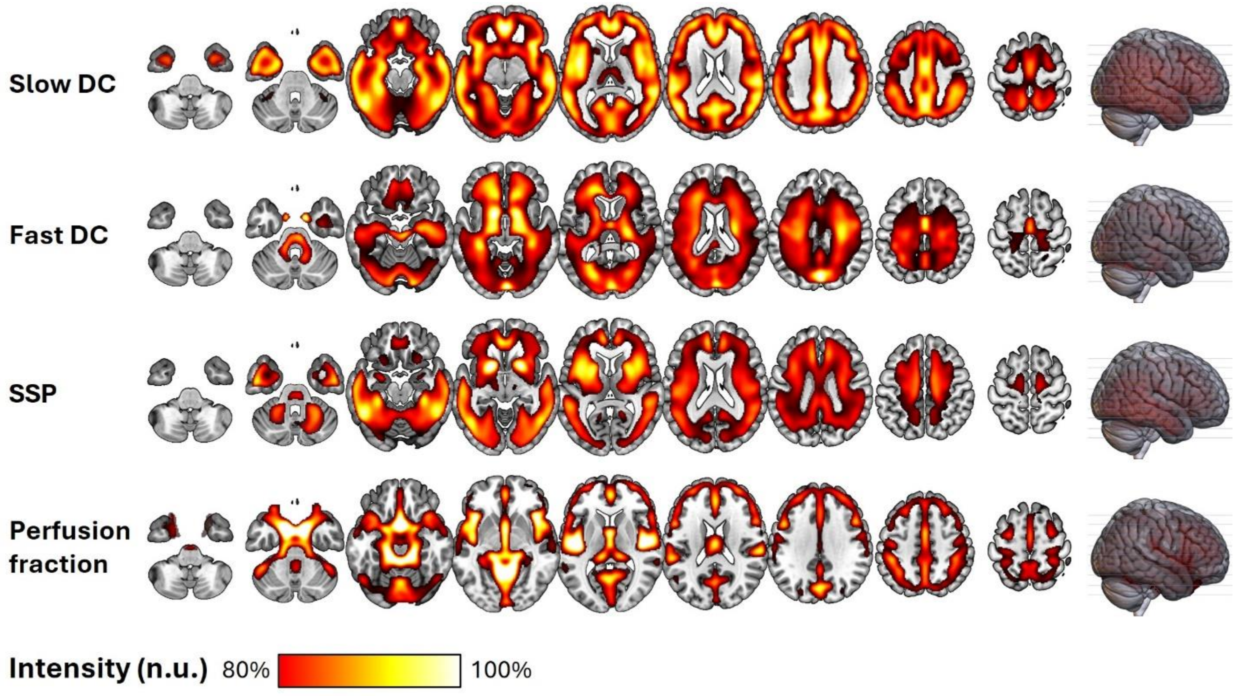

Supplementary Figure 1: Parameter maps derived with the three-compartment IVIM model, averaged across the A-T- subjects (n = 192)

The maps depict the regions exhibiting the 20% highest values for each of the IVIM parameters. The axial slices presented across the columns (left-right) are indicated by lines on the brain surface in the rightmost column (bottom-up).

DC: diffusion coefficient; nu: normalized units; IVIM : intravoxel incoherent motion; SSP: slow signal portion.

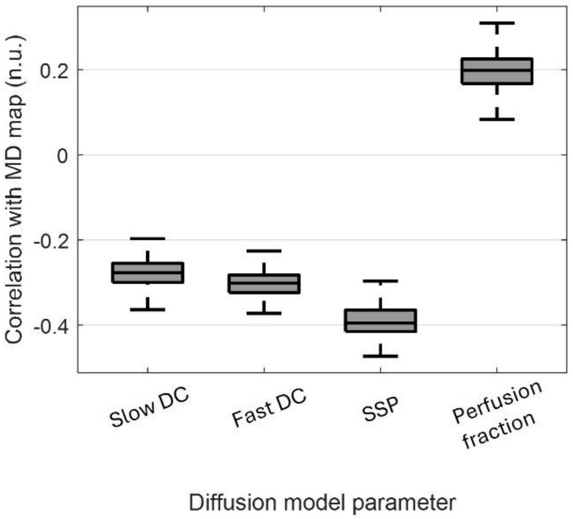

Supplementary Figure 2: Association between the IVIM and MD parameter maps in all subjects (n=297).

For each IVIM parameter map, the spatial variability across voxels in the GM and WM was correlated with the spatial variability in the MD map using Pearson correlation. Slow DC, fast DC, and SSP were found to be negatively correlated with MD, while the perfusion fraction was positively correlated with MD (p-value<0.001).

DC: diffusion coefficient; GM: gray matter; IVIM: intravoxel incoherent motion; MD: mean diffusivity; nu: normalized units; SSP: slow signal portion; WM: white matter.

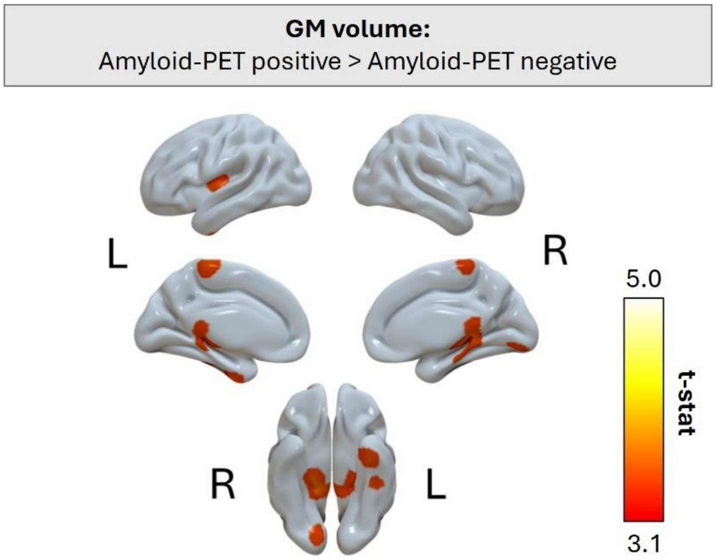

Supplementary Figure 3: Brain regions with GM volume positively associated with amyloid-PET positivity in all subjects with available amyloid beta PET (n=151).

Regions with significant associations between GM volume and amyloid-PET positivity were identified through multiple regression, considering GM volume as dependent variable, amyloid-PET positivity as covariate, and age, sex, and TIV as confounding variables. The brain maps depict regions with positive associations in red (i.e., higher GM volume linked to amyloid-PET positivity) based on *t*-stats with a threshold significance level of 0.005 (uncorrected for multiple comparisons; clusters smaller than 100 voxels were excluded). The axial slices presented across the columns (left-right) are indicated by lines on the brain surface in the rightmost column (bottom-up).

GM: gray matter; TIV: total intracranial volume.

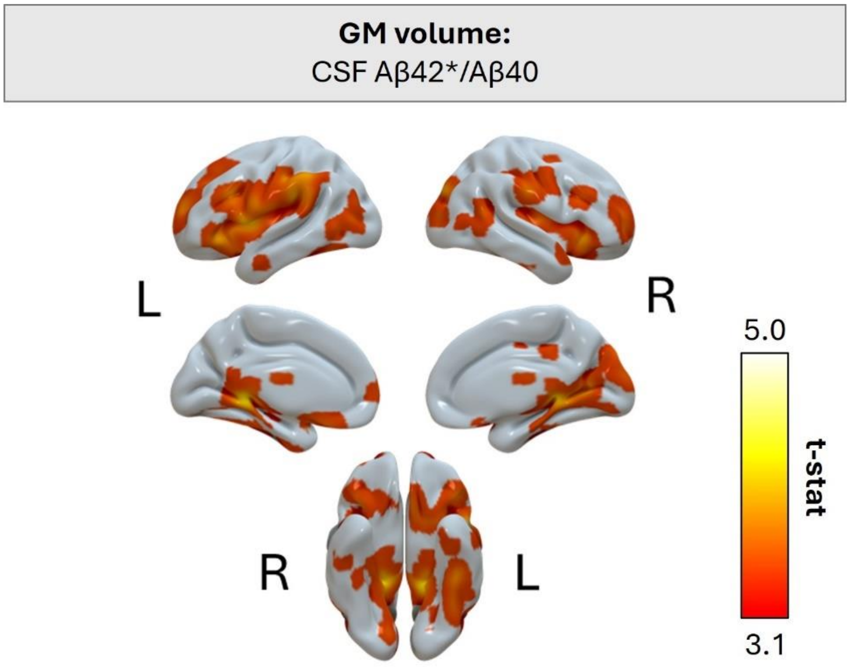

Supplementary Figure 4. Brain regions with GM volume negatively associated with CSF Aβ42/40 levels in all subjects (n=297).

Regions with significant associations between GM volume and CSF Aβ42/40 levels were identified through multiple regression, considering GM volume as dependent variable, CSF Aβ42/40 as covariate, and age, sex, and TIV as confounding variables. To align more closely with the analysis in the study by Salvadó *et al.*[11], we considered the CSF levels of Aβ42 measured with Elecsys®. The brain maps depict regions with negative associations in red (i.e., higher GM volume linked to lower CSF Aβ42/40 levels) based on *t*-stats with a threshold significance level of 0.005 (uncorrected for multiple comparisons; clusters smaller than 100 voxels were excluded). The axial slices presented across the columns (left-right) are indicated by lines on the brain surface in the rightmost column (bottom-up).

Aβ: amyloid beta; GM: gray matter; TIV: total intracranial volume.

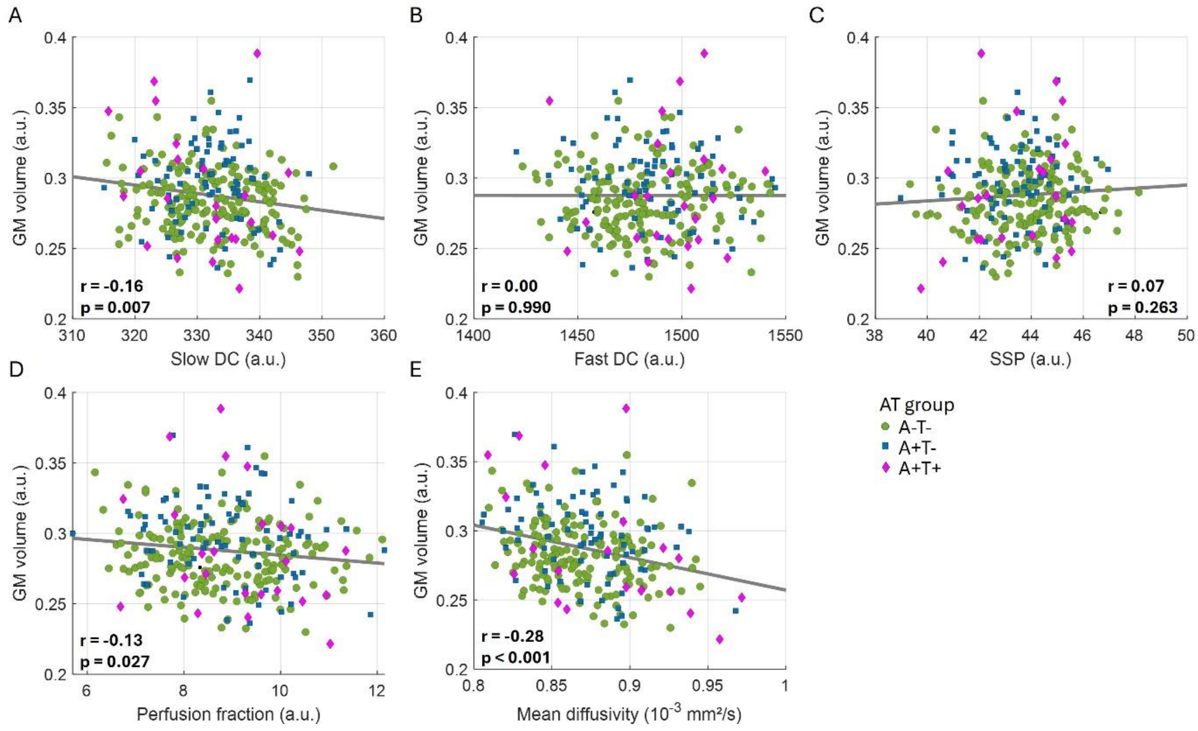

Supplementary Figure 5: Association between GM volume averaged across regions where GM volume was negatively associated with CSF Aβ42/40 levels and the diffusion parameters in all subjects (n=297).

Significant associations between GM volume and the diffusion parameters derived from the IVIM model, namely (A) slow DC, (B) fast DC, (C) SSP, (D) perfusion fraction, as well as (E) the MD diffusion parameter, were identified through multiple regression, considering each diffusion parameter as the independent variable, the GM volume as the dependent covariate, and age, sex, and TIV as confounding variables. Each data point represents a single participant. Interaction terms between diffusion parameters and AT stage were also tested in the regression model but were not statistically significant.

a.u.: arbitrary units; DC: diffusion coefficient; GM: gray matter; MD: mean diffusivity; SSP: slow signal portion.

References

1. Klunk WE, Koeppe RA, Price JC, et al. The Centiloid Project: Standardizing quantitative amyloid plaque estimation by PET. *Alzheimer's & Dementia*. 2015;11(1):1. doi:10.1016/j.jalz.2014.07.003

2. Shekari M, Vállez García D, Collij LE, et al. Stress testing the Centiloid: Precision and variability of PET quantification of amyloid pathology. *Alzheimer's & Dementia*. 2024;20(8):5102-5113. doi:10.1002/alz.13883

3. Friston KJ, Ashburner J, Kiebel SJ, Nichols TE, Penny WD, eds. *Statistical Parametric Mapping: The Analysis of Functional Brain Images*. Academic Press; 2007. http://store.elsevier.com/product.jsp?isbn=9780123725608

4. Puig-Pijoan A, García-Escobar G, Fernández-Lebrero A, et al. The CORCOBIA study: Cut-off points of Alzheimer's disease CSF biomarkers in a clinical cohort. *Neurología (English Edition)*. Published online August 9, 2022. doi:10.1016/j.nrleng.2022.05.002

5. Gispert JD, Rami L, Sánchez-Benavides G, et al. Nonlinear cerebral atrophy patterns across the Alzheimer's disease continuum: impact of APOE4 genotype. *Neurobiology of Aging*. 2015;36(10):2687-2701. doi:10.1016/j.neurobiolaging.2015.06.027

6. Zetterberg H, Bendlin BB. Biomarkers for Alzheimer's disease—preparing for a new era of disease-modifying therapies. *Mol Psychiatry*. 2021;26(1):296-308. doi:10.1038/s41380-020-0721-9

7. Milà-Alomà M, Salvadó G, Gispert JD, et al. Amyloid beta, tau, synaptic, neurodegeneration, and glial biomarkers in the preclinical stage of the Alzheimer's continuum. *Alzheimer's & Dementia*. 2020;16(10):1358-1371. doi:https://doi.org/10.1002/alz.12131

8. Milà-Alomà M, Brinkmalm A, Ashton NJ, et al. CSF Synaptic Biomarkers in the Preclinical Stage of Alzheimer Disease and Their Association With MRI and PET: A Cross-sectional Study. *Neurology*. 2021;97(21):e2065-e2078. doi:10.1212/WNL.0000000000012853

9. Salvadó G, Shekari M, Falcon C, et al. Brain alterations in the early Alzheimer's continuum with amyloid-β, tau, glial and neurodegeneration CSF markers. *Brain Communications*. 2022;4(3):fcac134. doi:10.1093/braincomms/fcac134

10. Suárez-Calvet M, Karikari TK, Ashton NJ, et al. Novel tau biomarkers phosphorylated at T181, T217 or T231 rise in the initial stages of the preclinical Alzheimer's continuum when only subtle changes in Aβ pathology are detected. *EMBO Molecular Medicine*. 2020;12(12):e12921. doi:https://doi.org/10.15252/emmm.202012921

11. Salvadó G, Shekari M, Falcon C, et al. Brain alterations in the early Alzheimer's continuum with amyloid-β, tau, glial and neurodegeneration CSF markers. *Brain Communications*. 2022;4(3):fcac134. doi:10.1093/braincomms/fcac134

12. Benedet AL, Milà-Alomà M, Vrillon A, et al. Differences Between Plasma and Cerebrospinal Fluid Glial Fibrillary Acidic Protein Levels Across the Alzheimer Disease Continuum. *JAMA Neurol*. 2021;78(12):1471. doi:10.1001/jamaneurol.2021.3671

13. Karlsson L, Vogel J, Arvidsson I, et al. *Cerebrospinal Fluid Reference Proteins Increase Accuracy and Interpretability of Biomarkers for Brain Diseases*. Neuroscience; 2023. doi:10.1101/2023.06.08.544222

14. Jack CR, Bennett DA, Blennow K, et al. NIA-AA Research Framework: Toward a biological definition of Alzheimer's disease. *Alzheimer's & Dementia*. 2018;14(4):535-562. doi:https://doi.org/10.1016/j.jalz.2018.02.018
